# Data set on interactive service quality in higher education marketing

**DOI:** 10.1016/j.dib.2018.05.082

**Published:** 2018-05-23

**Authors:** Olaleke Oluseye Ogunnaike, Banji Ayeni, Bankole Olorunyomi, Maxwell Olokundun, Omisade Ayoade, Taiye Borishade

**Affiliations:** a Covenant University, Nigeria; b Landmark University, Nigeria

**Keywords:** Interactive quality, Service marketing, University, Education marketing

## Abstract

This paper provides data on the interactive quality of the educational services rendered in south west, Nigeria. Data were gathered based on conclusive research design. Stratified and convenience sampling techniques were adopted. Responses were elicited from the alumni as regards to their perception towards the interactive quality; learning, group discussion, breadth, assignment, examination as well as social relationships. Interactive quality component of the Student Evaluation of Educational Quality (SEEQ) developed by previous scholars was adapted. The research instrument was confirmed to have all the necessary psychometric values considered appropriate for the study. Some descriptive statistical analyses were carried out to further clarify the data and provide the necessary platform for further analyses.

## 1 Introduction

The context determines the meaning of the word quality to different people. Quality can be described as conformance of output to planned goals, specifications and requirements [2]. Service marketing scholars believe that quality is about exceeding customer expectations [13]. quality in education as the fitness of educational outcome and experience for use [8]. This paper is premised on transcendent view of quality by Garvin [8]. Scholars argue that recognition of quality is dependent on experience gathered from repeated exposure to the service. This perspective of quality is consistent with innate excellence, high achievement and uncompromising standards [11], [12].

Interactive quality is one of the essential dimension of service quality. It refers to the nature of communication and relationship that exist between the students and faculty and staff of the University. It is also about the quality of teaching and learning process in the University. Instruction may be described as the impartation of skills, values as well as knowledge that came as a result of quality teaching. The education literature presents a good number of teaching strategies and there are also a good number of research studies that validate them [1], [10].

The issues of teaching quality and teaching effectiveness have been attracting scholarly debates and controversies in the higher education community. As a result, a good number of scholars focused on teaching quality from different views [6], [7]. Many researchers agreed to the fact that teaching quality is one of the major factors that influence student achievement, other school-related factors include financial condition, class size, leadership or school organization [3], [4]. However, only limited studies considered the views of the alumni of the universities [5].

**Specification Table**

**Table:** 

**Subject area**	Business, Education
**More Specific Subject Area:**	Education Marketing
**Type of Data**	Primary data, Tables
**How Data was Acquired**	Field survey
**Data format**	Descriptive statistical data
**Experimental Factors**	Purposive and convenience sampling techniques were used
**Experimental features**	Only graduates of the selected universities were sampled
**Data source location**	South west, Nigeria
**Data Accessibility**	Data are presented this article

**Value of data**

• The data, in this article, describe academic specialization of the students across the three categories of universities
• The results from these data can be used to assess the level of learning that took place in those universities
• It provides information on the quality of examination that take place in those universities as perceived by the students.
• The results of the data show the ratings of universities by their students as regards to the quality of assignments given, group discussion as well as the breadth of the knowledge being impacted on the students.
• The data can be used to compare the three categories of universities based on their perceived interactive quality. The results can further be categorised based on gender, academic specialization as well as state of origin
• Many studies have been done on technical and functional quality of higher institutions especially from the perspective of the regulatory bodies but limited studies have been done in the area of interactive quality. The data provided shall therefore facilitate further studies on interactive quality in higher education marketing.

## 2 Data

**Fig. 1:**
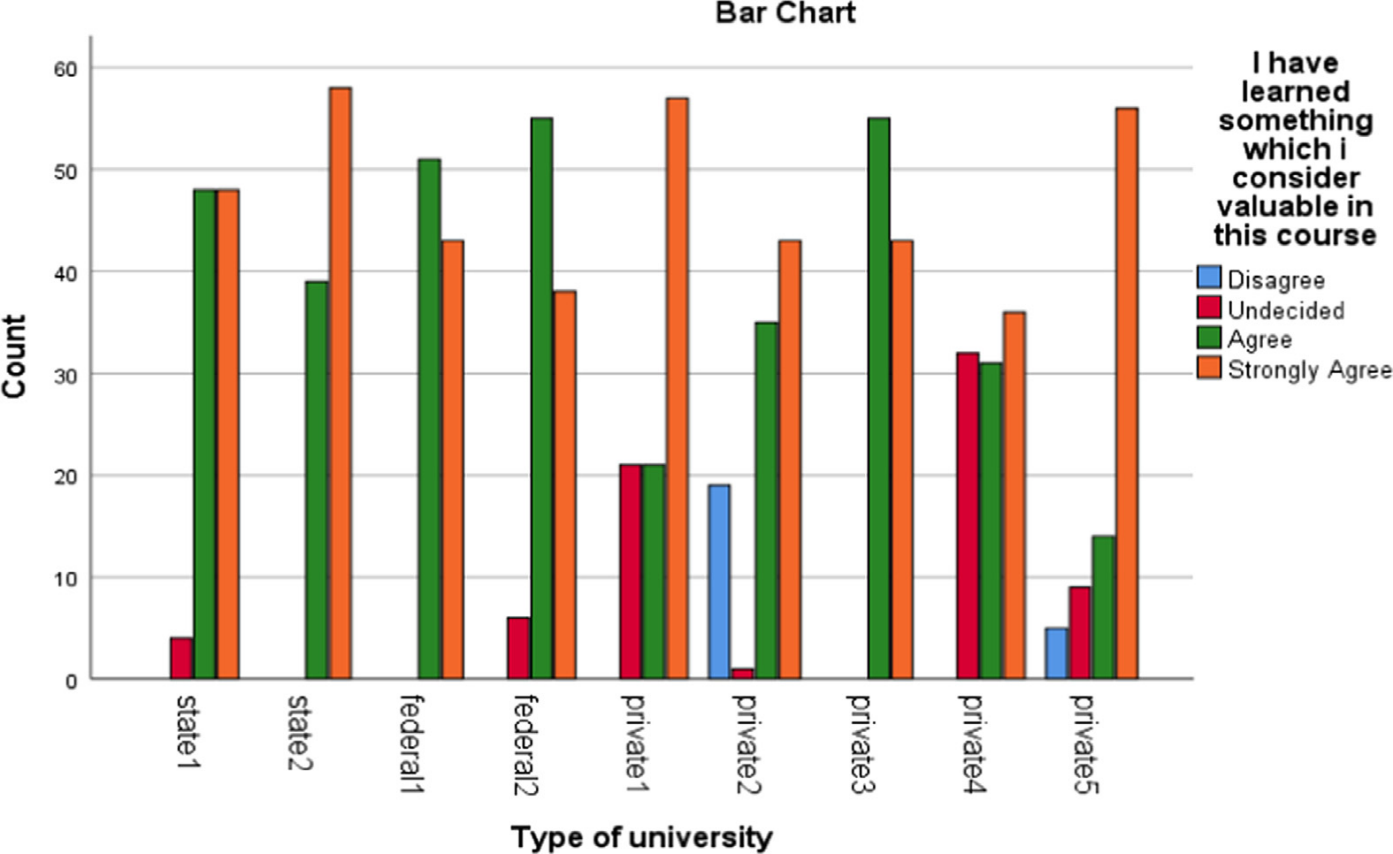
Learning Quality.

**Fig. 2:**
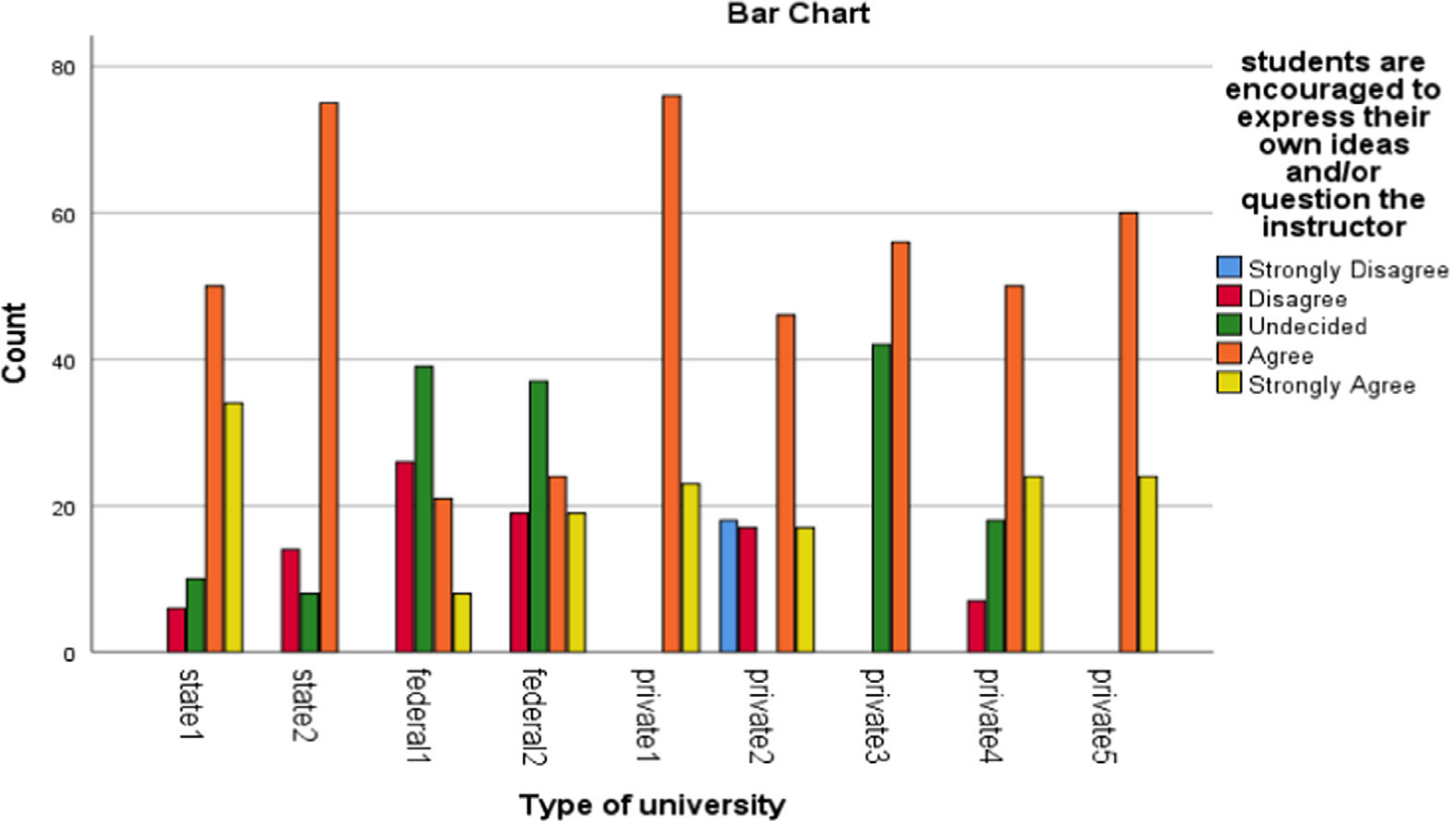
Group discussion.

**Fig. 3:**
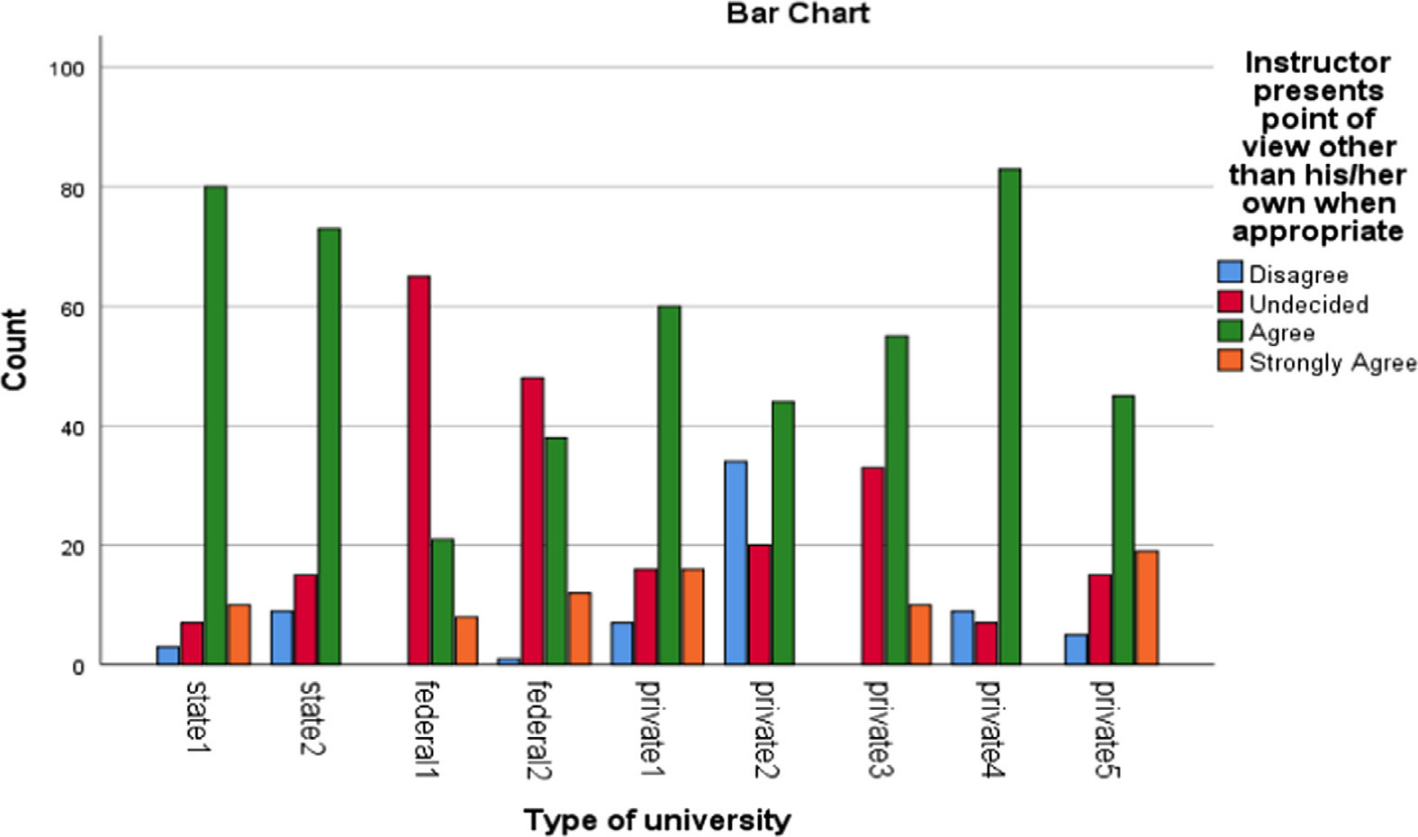
Breadth of the lecture.

**Fig. 4:**
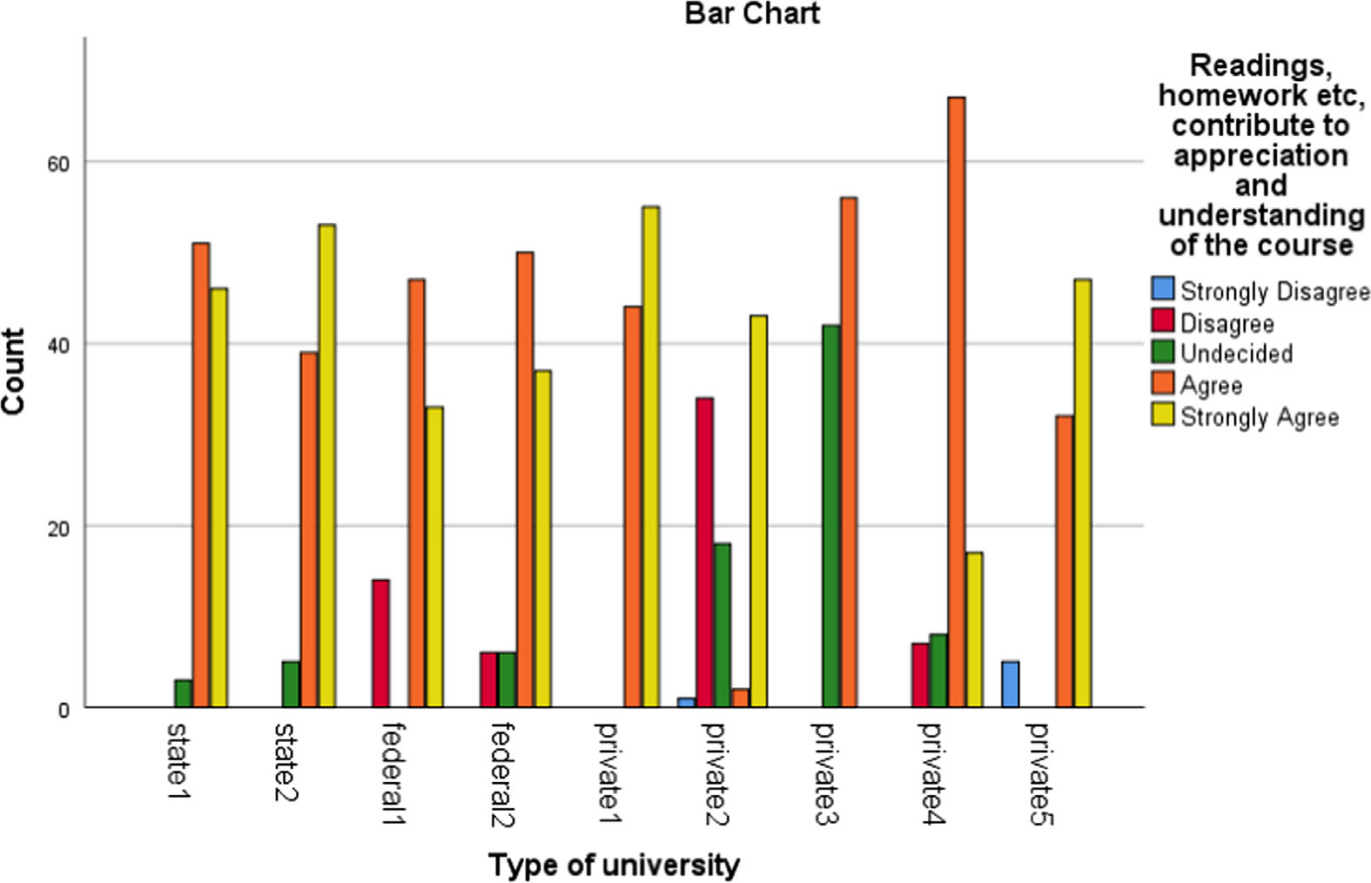
Assignment.

**Fig. 5:**
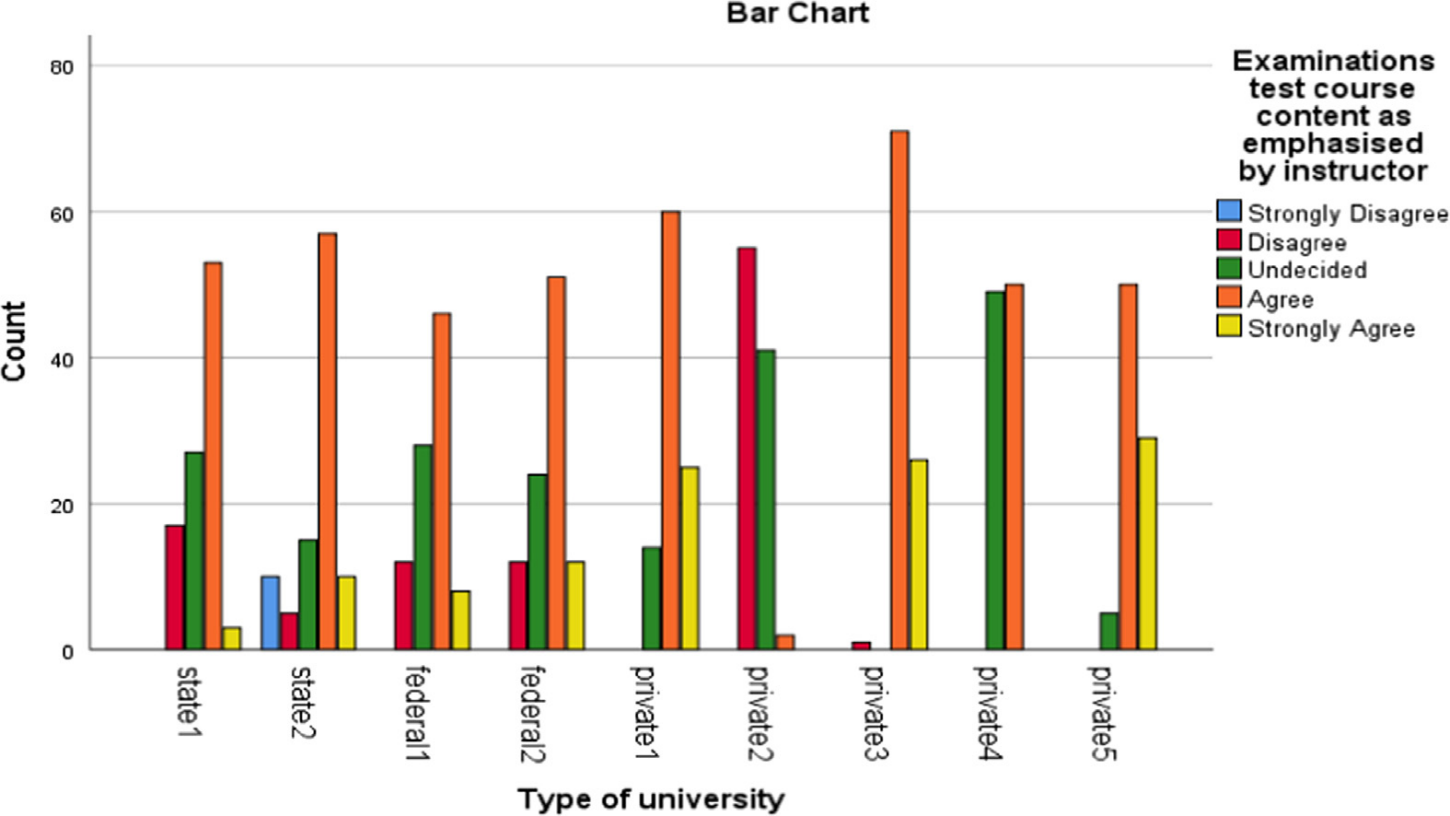
Examination.

**Fig. 6:**
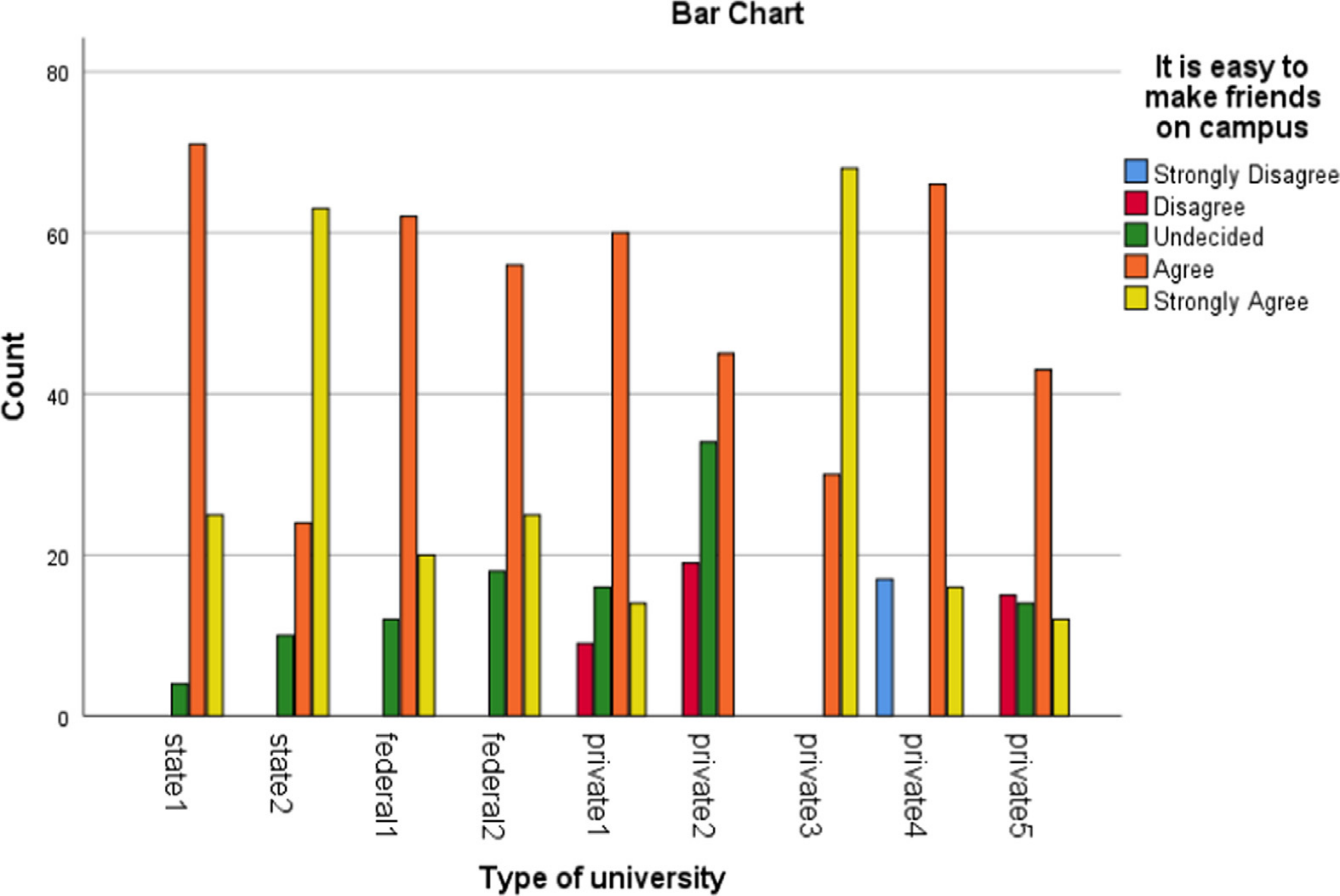
Social Relationship.

The data presented the academic disciplines of the respondents in the study. Industrial Relations and Human Resource Management, Accounting, Business administration as well as marketing that are captured as management related courses have the largest percentage of representation (33.2). The management related courses were mostly subscribed for in those Universities and as such the distribution is a good representation of the population. Other areas of specialization of the alumni and their corresponding percentages are social science courses (17.2%), science and environmental based courses (20.9%), Law (11.5%), Engineering courses(5.1%), education (6.1%) as well as art and humanities based courses (6.1%). Fig. 1, Fig. 2, Fig. 3, Fig. 4, Fig. 5, Fig. 6

### 2.1 Cross tabulation between interactive quality and type of quality

The alumni were asked to assess the interactive or instructional quality of their “alma matter”. The dimensions of interactive quality considered in this study included learning, group discussion, breadth of lecture, assignment, examination and social relationship. The bar charts below represented the extent to which these dimensions were rated by different categories of universities involved in this study.

The bar charts above revealed the variations in responses of the alumni of the universities involved in this study to the different dimensions of interactive quality.

## 3 Experimental design, materials and methods

This paper gathered data on interactive service quality imperatives among Nigerian Universities. Scholars have different opinions on the dimensions or components of service quality. Responses were elicited from the alumni to rate their universities based on their level of interactive quality; learning, breadth, assignment, group discussion, examination and social relationships. This paper considered the interactive component of service quality. The questionnaire was adapted from the works of previous scholars [9], [14].

In addition, the questionnaire was further subjected to factor analysis in order to ascertain its convergent validity. The result revealed that the least loading was 0.261 while the highest loading was 0.730. The adequacy of sampling was ascertained with KMO measure of 0.748 with Barlett׳s Test result of p=0.000. This result therefore suggests that the instrument pass the test of convergent validity.

## 4 Conclusion

This data article analyzed the responses of graduates of the selected universities in Nigeria as regards to the quality of interaction received during their undergraduate programmes. The data provided will encourage empirical studies that could assess the current trends in quality of education in Nigeria and how the education, as a service, could be improved upon and marketed to both internal and external stakeholders(customers).

It is hopeful that empirically based insights gathered from this data article will further contribute to relevant theories, policy formulation and practice in the academia.
