# Supplementary material for: Data set on interactive service quality in higher education marketing
Source: Data Brief. 2018 May 23;19:1403–9. doi: 10.1016/j.dib.2018.05.082 (PMC6141764; doi:10.1016/j.dib.2018.05.082)
Supplement: Supplementary file 1 — Supplementary material [file mmc1.zip › mmc1.docx]

Conflict of Interest

All the authors confirms no conflict of interest.
